# Oligo (Poly (Ethylene Glycol) Fumarate)-Based Multicomponent Cryogels for Neural Tissue Replacement

**DOI:** 10.3390/gels9020105

**Published:** 2023-01-25

**Authors:** Mohamed Zoughaib, Kenana Dayob, Svetlana Avdokushina, Marat I. Kamalov, Diana V. Salakhieva, Irina N. Savina, Igor A. Lavrov, Timur I. Abdullin

**Affiliations:** 1 Institute of Fundamental Medicine and Biology, Kazan (Volga Region) Federal University, 18 Kremlyovskaya St., 420008 Kazan, Russia; 2 Scientific and Educational Center of Pharmaceutics, Kazan (Volga Region) Federal University, 18 Kremlyovskaya St., 420008 Kazan, Russia; 3 School of Applied Sciences, University of Brighton, Huxley Building, Lewes Road, Brighton BN2 4GJ, UK; 4 Department of Neurology, Mayo Clinic, Rochester, MN 55905, USA

**Keywords:** oligo (poly (ethylene glycol) fumarate), multicomponent cryogels, polyethylene glycol, cationic monomer, physicochemical properties, porous structure, neuronal cells, cell-supporting properties

## Abstract

Synthetic hydrogels provide a promising platform to produce neural tissue analogs with improved control over structural, physical, and chemical properties. In this study, oligo (poly (ethylene glycol) fumarate) (OPF)-based macroporous cryogels were developed as a potential next-generation alternative to a non-porous OPF hydrogel previously proposed as an advanced biodegradable scaffold for spinal cord repair. A series of OPF cryogel conduits in combination with PEG diacrylate and 2-(methacryloyloxy) ethyl-trimethylammonium chloride (MAETAC) cationic monomers were synthesized and characterized. The contribution of each component to viscoelastic and hydration behaviors and porous structure was identified, and concentration relationships for these properties were revealed. The rheological properties of the materials corresponded to those of neural tissues and scaffolds, according to the reviewed data. A comparative assessment of adhesion, migration, and proliferation of neuronal cells in multicomponent cryogels was carried out to optimize cell-supporting characteristics. The results show that OPF-based cryogels can be used as a tunable synthetic scaffold for neural tissue repair with advantages over their hydrogel counterparts.

## 1. Introduction

Being a severe and widespread form of neuronal trauma, spinal cord injury (SCI) represents a challenging medical problem with a high impact on patient physical ability, quality of life, and the cost of treatment and rehabilitation [1,2]. Currently available therapies for SCI often demonstrate modest recovery without achieving complete restoration of motor and sensory functions [2,3]. 

Biomaterial implants prove to be an important tool to replace damaged neural tissues and promote their regeneration per se or in combination with other treatments [4]. Given that the SCI site lacks extracellular matrix (ECM), the implanted scaffolds should fill the lesion gap, providing ECM-mimicking support for cell survival and neurite outgrowth. To further stimulate the repair of neural tissues, the scaffolds can be modified with cell-instructive biochemical and physical signals and/or loaded with bioactive products such as donor cells and drug molecules [5,6].

Currently employed biomaterials for the treatment of SCI are summarized in recent reviews [4,7,8,9]. Natural polymers, such as collagen [10], gelatin [11], hyaluronic acid [12], fibrin [13], and alginate [14], are well established structure-forming components of tissue-engineered materials for SCI repair. In spite of their biocompatible and biodegradable nature with partially preserved ECM functionalities (both cell-supporting and immunogenic), natural polymers commonly do not provide precise control over the (bio)chemical, structural, and physicochemical properties of the resulting scaffolds, which is particularly important for the treatment of injury at different parts of the central nervous system (CNS) [14,15]. In this regard, synthetic polymer materials, such as poly(ethylene glycol) (PEG) [16], poly(2-hydroxyethyl methacrylate) (pHEMA) [17], poly(lactic co-glycolic acid) (PLGA) [18], and poly(ε-caprolactone) (PCL) [19], have several benefits, including high purity, low immunogenicity, and amenability to various modifications as well as tuning biochemical and mechanical characteristics [7,15,20].

Two of the proposed biodegradable materials have been approved for clinical trials in patients with SCI, namely, Neuro-Spinal, a porous scaffold composed of PLGA-poly(L-lysine) [21] and NeuroRegen, a decellularized collagen scaffold loaded with mesenchymal stem cells [22]. Due to the low to moderate therapeutic outcomes of these materials, new products are still in high demand, which ideally should ensure effective posttraumatic recovery of the spinal cord while having compliance with large-scale production, sufficient stability, and an extended shelf-life [23,24].

OPF-based hydrogels have been shown to afford a permissive regenerative environment to repair SCI, providing scaffolding for axon regeneration [25,26,27]. Based on these auspicious effects, a positively charged oligo (poly (ethylene glycol) fumarate) (OPF) hydrogel was recently proposed by Siddiqui et al. as a tissue-replacing scaffold in SCI with a set of longitudinal channels introduced to the hydrogel to facilitate and guide axonal growth, bridging damaged nerve fibers [1]. The material was seeded with neurotrophin-producing Schwann cells and PLGA microspheres loaded with rapamycin to inhibit glial scar formation. Combined treatment with the OPF-based scaffold and epidural electrical stimulation allowed the cumulative enhancement of motor function improvement in rats with complete spinal transection [1]. 

Whereas the above-mentioned scaffolds are based on non-porous hydrogels with mechanically formed channels, it is of substantial interest to evolve these materials by developing their macroporous analogs prepared by the cryogelation technique. The corresponding cryogels feature a unique interpenetrating porous structure that facilitates the mass transfer of gases and nutrients and supports cell infiltration and three-dimensional growth, which are commonly unachievable in conventional hydrogels [28,29]. Furthermore, the structure of cryogels allows for in situ bulk modification of the scaffold with bioactive components, ensuring their increased availability for biointeractions [30].

In this study, we describe for the first time the design and synthesis of OPF-based cryogels as a potential next-generation of OPF scaffolds for the treatment of SCI. Since the implantable scaffold should primarily possess balanced characteristics of stiffness and softness in order to be integrated with nerve fibers while avoiding deformation by surrounding tissues [20,31], multi-component cryogels with tunable composition and mechanical properties were produced. The important role of the combination of an OPF macromer and a bifunctional PEG cross-linker in the formation of a well-structured polymer network and the regulation of both viscoelastic properties and water content in macroporous cryogels was established. The composition of OPF, PEG, and MAETAC cationic monomers was optimized to support the neural cells in the matrix. The obtained cryogels provide enhanced cell adhesion, migration, and proliferation and serve as a platform for the development of biomaterial candidates for preclinical studies.

## 2. Results

### 2.1. Synthesis of OPF-Based Cryogels

**Figure 1:**
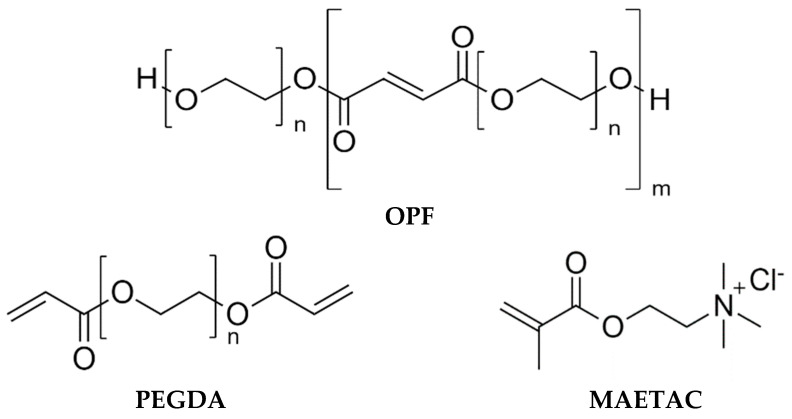
Chemical structures of cryogel constituents: oligo (poly (ethylene glycol) fumarate) (OPF), polyethylene glycol diacrylate (PEGDA), and 2-(methacryloyloxy)ethyl-trimethylammonium chloride (MAETAC).

The OPF macromer was synthesized in the reaction of PEG (1500 Da) with fumaryl chloride (Section 4.2). The FTIR spectrum of the OPF macromer, in addition to the typical C–O signal of the ether bond in PEG (1100 cm^−1^), contained the peaks at 1730 and 1656 cm^−1^, which were respectively attributed to the C=O and C=C groups of the fumaryl moiety (Figure S1). The presence of C=O and C=C groups in the synthesized macromer was also confirmed by ^1^H and ^13^C–{^1^H} NMR spectroscopy (Figure S2). This analysis verifies the co-polymerization of PEG and fumaryl moieties to form OPF chains (Figure 1) capable of radical polymerization and biodegradation [32]. Based on the regression relationship between the molecular weight (MW) of the PEG precursor and the OPF product [33], the MW of the synthesized macromer was estimated to be ca. 29 kDa.

A series of macroporous cryogels, composed of OPF, PEG diacrylate (PEGDA), and MAETAC (Figure 1), were synthesized, as detailed in Section 4.3, with variable concentrations of the constituents. The prepared materials were divided into four groups: A–D, where the concentration of one component was varied in each group (Table 1). The difference in composition affected the appearance of the obtained cryogels, as shown in Table 1. The as-prepared cryogels were in the form of cylindrical products with diameters ranging from 0.9 to 1.3 mm depending on their swelling ability. 

According to an FTIR analysis data, multicomponent OPF-derived cryogels were characterized by an increased C=O signal (1730 cm^−1^) relative to the reference C–O signal and the absence of C=C groups. The MAETAC constituent generated a new peak at 950 cm^−1^ attributed to the quaternary ammonium group. The ratio between the peaks at 950 and 1100 cm^−1^ almost linearly increased with the MAETAC concentration in the reaction solution, indicating a proportional incorporation of the cationic monomer into the polymer chains of the cryogels (r^2^ = 0.93, Figure S1).

### 2.2. Rheological Behavior

**Figure 2:**
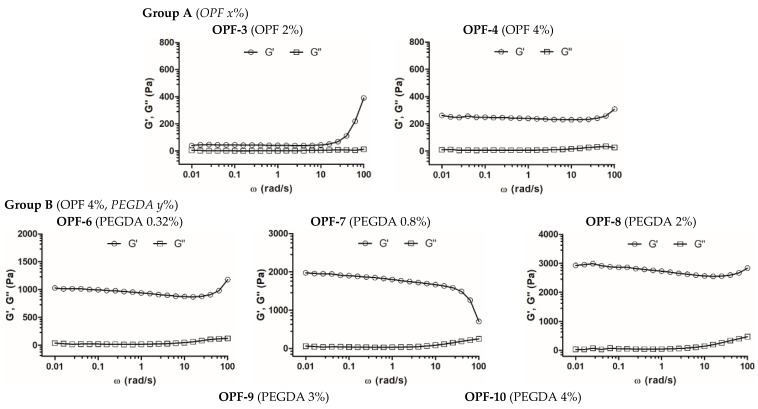

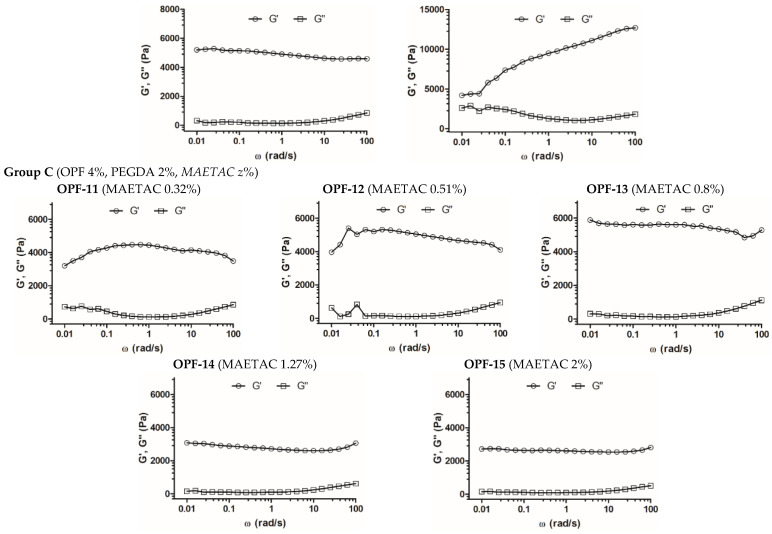
Frequency sweep analysis of OPF-based cryogels. The measurement of frequency dependence of storage (G′) and loss (G″) modulus was performed within LVR at δ = 0.5% strain deformation. Cryogel compositions (wt. %) are shown in parentheses.

Viscoelastic properties of the synthesized cryogels were analyzed by small-amplitude oscillatory shear measurements within the linear viscoelastic region, observed at the shear strain amplitude (γ) ≤ 1%. Both the storage (G′) and the loss (G″) modulus of most of the studied materials depended relatively weakly on the frequency in the range of up to 10 rad s^−1^, whereas G′ strongly prevailed over G″ (Figure 2, Table 2), which indicates the formation of a stable and well-structured hydrogel network with a dominant elastic behavior [34]. The contribution of constituents to the mechanical properties of cryogels was assessed.

For the formation of one-component OPF cryogels (group A), a critical concentration of OPF ≥ 2 wt.% was required. Materials of this group with an OPF concentration of 2–6 wt.% were quite soft and difficult to handle compared to the cryogels of other groups. The cryogel with 4 wt.% OPF had a higher G′ value (ca. 240 Pa) than that with 2 wt.% OPF (40 Pa), while the G′/G″ ratio for both materials was similar (Figure 2, Table 2, OPF-3, and OPF-4). Furthermore, at a concentration of 6 wt.%, part of the OPF was not entrapped and was washed out of the material; therefore, this cryogel was not studied further. The results show the relatively poor ability of the OPF macromer alone to produce a stable polymer network in the cryogel system, which is attributed to the restricted availability of the C=C reactive group in the fumaryl moieties, which are surrounded by PEG fragments. The additional cross-linking of the polymer network with a bifunctional PEG derivative PEGDA improved incorporation of the OPF macromer and increased gel strength (groups B–D). 

However, the OPF cryogels with 4 wt.% OPF and upper concentrations of PEGDA 3 and 4 wt.% (OPF-9 and OPF-10) were characterized by a reduced G′/G″ ratio. Moreover, for the OPF-10 material, the G′ and G″ parameters began to strongly depend on frequency (Figure 2, Table 2). This altered viscoelastic behavior can be attributed to a certain inhomogeneity of the material due to the flocculation of the excess PEG component, which is promoted by a subzero temperature.

In addition, the effect of MAETAC on cryogels composed of 4 wt.% OPF and 2 wt.% PEGDA was evaluated (group C). The elasticity of these materials increased moderately and linearly (r^2^ = 0.94, Figure S3), with a MAETAC concentration of up to 0.8 wt.% (Figure 2, Table 2), indicating the contribution of the monomer to the formation of the polymer network.

At a higher amount of MAETAC (1.3–2 wt.%), such a promoting effect generally disappeared, which was also accompanied by some decrease in the G′/G″ ratio. This can be explained by a critical concentration of the monomer at ca. 0.8 wt.%, above which increased cationization and electrostatic repulsion of polymer chains occur, which affect the cross-linking degree of the resultant polymer network.

The analysis of the group D data demonstrated that in the absence of PEGDA, 0.8 wt.% MAETAC weakly contributed to the viscoelastic properties of the OPF material, indicating that the PEGDA component is necessary to produce stable multicomponent gels (Figure S5, Table 2).

### 2.3. Swelling Behavior

The swelling properties and porous structure of cryogels were assessed and analyzed in correlation with the rheological data. The cryogel network contains both weakly bound capillary water (CW) and polymer-bound water (PW). As demonstrated previously, the relative amounts of CW and PW in as-prepared cryogels can be differentiated [35]. The CW present in large pores can be readily removed by absorption with filter paper, while the PW is retained in the polymer network (pore ‘walls’) due to bonding with the polymer molecules. The relative amounts of CW and PW (%) in fully hydrated cryogels were estimated in correlation with porosity and cross-linking density.

**Figure 3:**
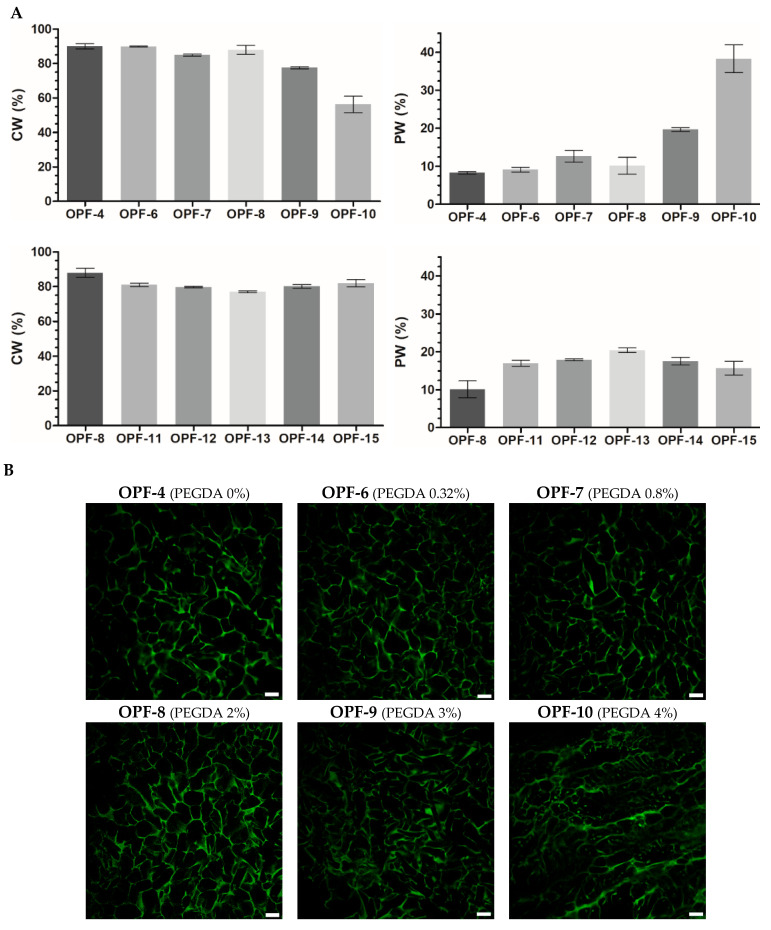

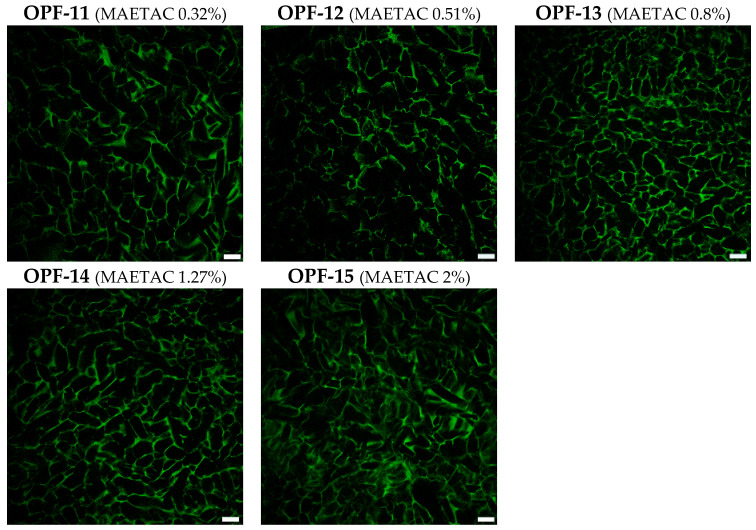
(**A**) The swelling properties of OPF cryogels (OPF 4 wt.%) with an increased amount of PEGDA (OPF-4–OPF-10) or OPF/PEGDA cryogels (OPF 4 wt.%, PEGDA 2 wt.%) with an increased amount of MAETAC (OPF-11–OPF-15). The mass content of capillary water (CW) and polymer-bound water (PW) is shown (mean ± SD, *n* = 3). (**B**) LSCM images of OPF cryogels are visualized by autofluorescence upon argon laser excitation (514 nm). The variable amounts (wt.%) of PEGDA and MAETAC are shown in parentheses. Scale bar 100 µm.

The PEGDA constituent, generally in proportion to its added concentration, was found to decrease the amount of CW and increase the amount of PW in OPF cryogels (Figure 3A). In consistency with that, laser scanning confocal microscopy (LSCM) visualization of the materials showed that the addition of PEGDA significantly reduced the size of macropores (Figure 3B) filled with CW. Similar changes appeared in gelatin-based cryogels upon their additional cross-linking by divalent metals [35]. 

The introduced MAETAC decreased the CW and increased the PW of OPF/PEGDA cryogels at a monomer concentration of 0.3–0.8 wt.%, above which the effect was partially reversed similarly with the concentration dependence of G′ (Figure 2). All the OPF-based materials were characterized by a well-organized macroporous structure; however, at a higher amount of both PEGDA (OPF-10) and MAETAC (OPF-15) constituents, the polymer walls and macropores of the cryogels appeared less defined and less homogenous (Figure 3). Such a modulation of the cryogel structure was accompanied by a decrease in the G′ and G′/G″ ratios (Figure 2).

Together, these results reveal clear relationships between rheological and hydration properties as well as the porous structure of the cryogels, allowing for better characterization of the produced materials. These also indicate that CW and PW differentiation provides a more informative structure characterization of the materials compared to the overall swelling index (Figure S4). In accordance with that, the equilibrium swelling values of macroporous cryogels were found to be apparent and inapplicable for the precise assessment of the materials [36]. Furthermore, based on the relative mass content of CW and PW in the swollen cryogels, the percent volume of macropores can be roughly estimated [35]. This parameter varied between 78 and 92% for the studied cryogels (Figure 3).

### 2.4. Cell Behavior in OPF Cryogels

#### 2.4.1. Cell Migration

Rat pheochromocytoma PC-12 and human neuroblastoma SH-SY5Y cells were used as established neuronal cells to assess biomaterials [37]. Among the pre-optimized methods of cell introduction into the cryogels [38], top seeding was used to study cell migration, adhesion, and proliferation in the materials.

**Figure 4:**
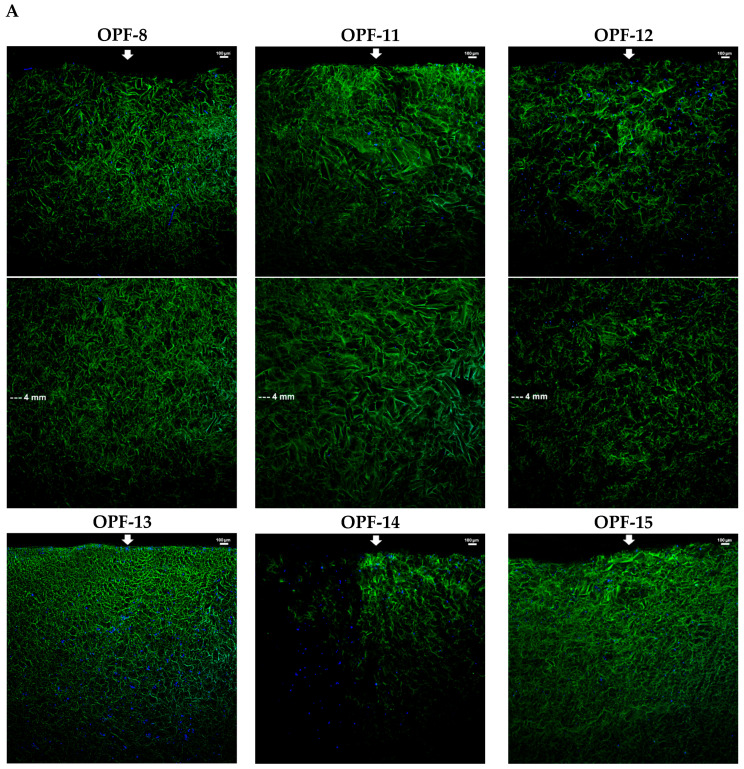

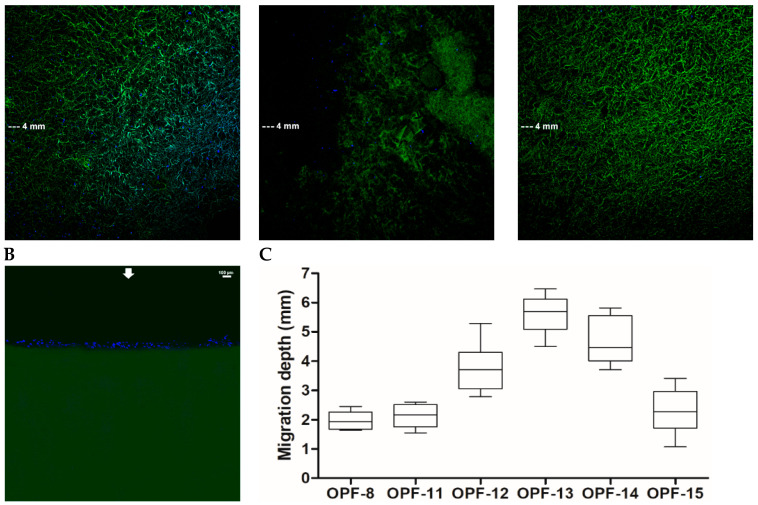
LSCM microphotographs of the lateral sections of OPF cryogels with a variable MAETAC amount (**A**) and an OPF hydrogel (**B**) with PC-12 cells migrating towards culture medium. The cells were incubated for 6 h on top of the materials mounted over the medium, which were fixed and stained with DAPI. The lateral sections of each cryogel were combined from the upper and lower parts; the arrows indicate cell seeding and migration sides. (**C**) The mean depth of cell migration into cryogels was shown.

The materials with 4 wt.% OPF, 2 wt.% PEGDA, and a varied amount of MAETAC were selected for a cell migration assay. The cryogels were immersed in a full culture medium to their half-height (5 mm). After equilibration with the medium, PC-12 cells were seeded on top of the materials and allowed to migrate down along the conduits towards a medium gradient. The results showed that the cells readily infiltrated cryogels and that adding MAETAC to the cryogel composition considerably promoted cell migration. In particular, the highest migration efficiency was observed for an intermediate MAETAC concentration of 0.8 wt.% (OPF-13), where the cells penetrated to a depth of 5.6 ± 0.7 mm. At a higher monomer concentration of 2 wt.% (OPF-15), the process was hampered almost to the level of MAETAC-free material (Figure 4). 

The results demonstrate that MAETAC supports cell migration in OPF cryogels in the optimal concentration range, above which the materials appear to acquire excess cationic charge. Interestingly, exceeding the optimal concentration (0.8 wt.%) was accompanied by a distinctive transition in the viscoelastic properties of materials (Figure 2).

In contrast to the above cryogels, the OPF hydrogel analog, regardless of the presence of MAETAC, did not support the infiltration of cells, so that they remained completely on the upper surface of the material (Figure 2). This demonstrates that the developed porous structure of the cryogel scaffold ensures increased cell migration, which should be due to both the spatial availability of macropores for cell penetration and a better exchange process between the material and medium, providing a decreased gradient of nutrients. Such cell-supporting properties are essential for material integration with host tissues and stimulation of regeneration-related processes, especially in the earlier stages prior to neovascularization [39,40].

#### 2.4.2. Cell Adherence and Proliferation

**Figure 5:**
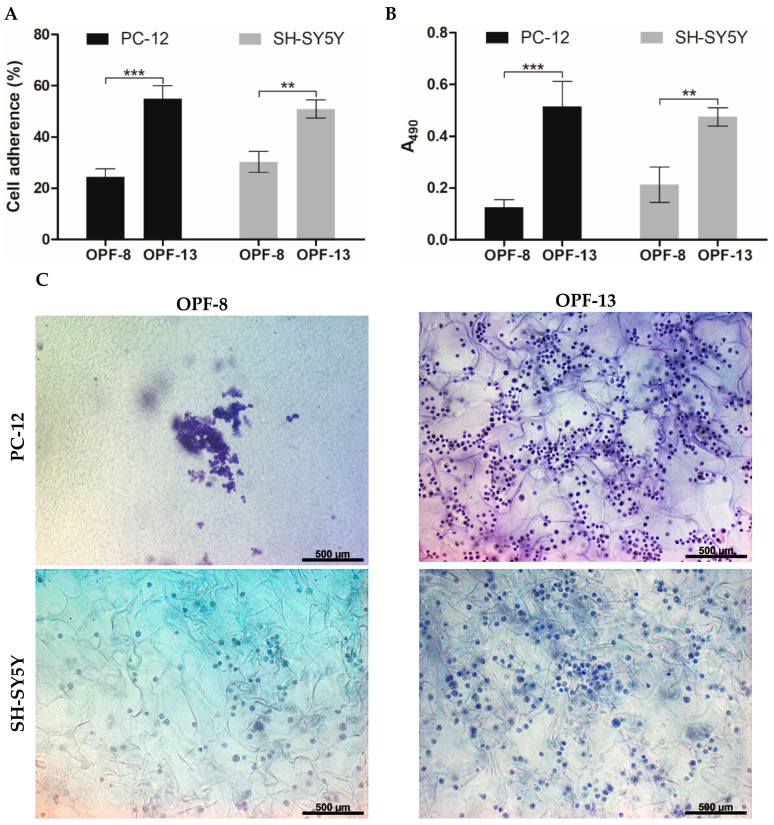
(**A**) Cell adherence on OPF cryogels after 4 h of incubation (% relative to the total number of seeded cells). (**B**) Proliferation of PC-12 and SH-SY5Y on OPF cryogels determined by an MTS assay at day 3 post-seeding. The data are presented as mean ± SD (*n* = 3, ** *p* < 0.01, and *** *p* < 0.001). (**C**) Representative bright-field microscopy images of PC-12 and SH-SY5Y cells, fixed and stained with cresyl violet at day 3 post-seeding on OPF cryogels.

The cell-supporting properties of the optimized OPF-13 cryogel were further evaluated. Compared to the MAETAC-free material (OPF-8), the OPF-13 material provided a high primary adhesion of both PC-12 and SH-SY5Y cells with an adhesion rate of 55 and 51%, respectively (Figure 5A), where the cationic monomer profoundly contributed to cell attachment, increasing it by 2.3 and 1.7 times, respectively. According to the MTS assay (72 h), MAETAC promoted cell proliferation within the OPF-13 cryogel by 4.1 (PC-12) and 2.2 (SH-SY5Y) times over the MAETAC-free OPF-8 cryogel (Figure 5B).

The latter results were in agreement with the bright-field microscopy analysis of the cells in the matrix stained with cresyl violet (Figure 5C). In contrast to the OPF-8 cryogel, the OPF-13 cryogel showed an increased number of cells of both types. SH-SY5Ycells were well distributed on both materials, with a mean cell number of 13 ± 4 (OPF-8) and 30 ± 9 (OPF-13) per mm^2^ of cryogel section. PC-12 cells tended to form cell ensembles on the OPF-8 cryogel, complicating their counting, whereas they appeared as multiple individual cells on the OPF-13 cryogel (39 ± 7 per mm^2^ of cryogel section), indicating a higher requirement of the cationic charge by these cells to spread and grow in the matrix (Figure 5C). 

Collectively, the results show that positively charged OPF cryogels that incorporate a MAETAC monomer allow superior cell attachment and distribution over MAETAC-free cryogels, thus providing an increased cell population of the materials.

## 3. Discussion

In this study, the structural and mechanical properties of OPF-based cryogels were analyzed depending on the concentrations of OPF, bifunctional PEGDA, and cationic MAETAC constituents. The results show that the material stiffness can be modulated in a controllable manner by varying the amount of PEGDA added to the OPF solution. The elastic modulus of the OPF cryogel profoundly (up to 40 times) and gradually increased with increasing PEGDA concentration. However, at a critical value of 3 wt.% PEGDA (75% by weight of OPF), the polymer network of cryogel appeared to be less organized, presumably due to some kind of phase separation at an excessive concentration of PEG.

Compared to PEGDA, MAETAC has relatively little effect on the mechanical properties of the materials composed of both OPF and OPF/PEGDA. The latter cryogel system became altered when the MAETAC concentration was ≥1.3 wt.% (22% of the total weight of OPF and PEGDA) (Figure 2, Table 2). Taken together, these results suggest working concentration ranges for PEGDA and MAETAC to obtain homogeneous multi-component OPF-derived cryogels with tunable viscoelastic properties. Previously, non-porous OPF hydrogels modified with PEGDA [41] or MAETAC [42] alone had been prepared. However, concentration-dependent transitions of their elastic modulus have not been reported to compare them with our data for cryogels. 

The observed changes in the mechanical properties of OPF cryogels containing PEGDA and MAETAC are consistent with changes in the water content and the porous structure of the swollen materials (Figure 3). In particular, an increase in G′ and G″ values was generally observed, along with a reduction in both the CW and the size of macropores and an increase in the PW. This can be explained by the formation of a more developed polymer network in the multi-component materials, which gives them better viscoelastic properties and water retention within the polymer chains. However, it should be expected that additional swelling of the polymer network will be accompanied by a shrinkage of macropores. This indicates that information on the CW and PW content in cryogels can complement their rheological characteristics, providing insights into the organization of the polymer component of materials.

Given that the mechanical properties of biomaterial scaffolds play an important role in their regenerative ability towards CNS, the literature data on the viscoelastic parameters of the SC and brain in mammals were reviewed. There is some evidence that CNS explants are soft, with an elastic modulus of 330 Pa [43] and ca. 100–600 Pa [44]. Higher G′ values for in situ/preconditioned rat brains were also reported to be as follows: 3336/1754 Pa (immature) and 1721/1232 Pa (old) [45]. A comparative evaluation of the previously reported data is complicated since the behavior of CNS tissues strongly depends on specimen characteristics, age, and analysis conditions [46]. Although rheological analysis has become a standard technique to characterize gels, different methods have been used to assess the viscoelastic parameters of living tissues, including brain and SC explants [44,46,47]. The rheological data summarized in the review [46] include quite variable G′ values for CNS, ranging from ca. 0.2 to up to 12 kPa.

Furthermore, no clear consensus exists on the optimal elastic and loss moduli for SC-replacing scaffolds. Table 3 summarizes the corresponding data on representative gels composed of natural and synthetic constituents. For some materials, G′ values were generally adjusted below 1 kPa [23,48,49,50,51,52,53], whereas in other studies, stiffer materials with G′ values up to several kPa were preferred [54,55,56,57,58]. It was suggested that softer hydrogels (G′ < 1 kPa) could be advantageous in supporting adhesion, proliferation, and differentiation of neural stem cells in vitro [59]. Similar results were obtained for CNS-derived cells encapsulated in three-dimensional hydrogel scaffolds [60] or cultured on their surfaces [43,61], whereas stiffer gels (G′ is ca. 10 kPa) somewhat inhibited cell functions [61]. 

Other evidence shows that neural stem cells exhibit increased proliferation and differentiation on PEG-based hydrogels with G′ values between 3.5 and 5.5 kPa [62]. In vivo applications should provide additional requirements on the mechanical properties of implantable hydrogels since insufficient stiffness impairs the integration of a scaffold with surrounding tissues and may result in its deformation and pore collapse [63]. It is also worthy of noting that many studies on the scaffolds rely on the G′/G″ values of CNS explants recited from papers, which do not deal with primary experimental data, further complicating the selection of optimal parameters.

The optimized multi-component cryogels composed of OPF (4 wt.%), PEGDA (2 wt.%), and MAETAC had an elastic modulus of up to ca. 5.5 kPa, which can be considered close to the upper value based on the reported data for CNS-replacing hydrogels. One can expect that the optimal parameters for the cryogels should differ from those for conventional hydrogels since most of their volume is occupied by interconnected macropores that are filled with mobile water. Due to the presence of low-density macropores, the cryogels should be stiffer to maintain their shape compared to hydrogels, especially upon implantation. Extra stiffness of cryogel materials could also be required to extend their design to SC conduits with hollow channels to promote nerve guidance [65] as well as to consider the softening of biodegradable conduits in vivo [66]. Based on the foregoing, the exact mechanical properties of OPF-based cryogels will be established afterwards depending on the final formulation and in vivo conditions, and these could be adjusted in a controllable manner by varying the concentration of PEGDA and other constituents.

In addition to having tunable structural properties, the multi-component cryogels were proven to serve as an effective scaffold for neuronal cells (Figure 4 and Figure 5). The materials supported the bulk migration and proliferation of the cells, which were not achieved for the hydrogel counterparts as previously observed for gelatin-based materials [38]. The prevailing interconnected macropores obviously improve cell infiltration and growth in different parts of the scaffold by promoting cell and nutrient penetration into the materials [28]. Even when channeled, the hydrogels are not expected to ensure such processes, which are important to promote angiogenesis and other regeneration-related events in the whole material [67]. 

Furthermore, the optimal cell-supporting concentration of MAETAC in the cryogels was revealed (Figure 4 and Figure 5). The selected OPF-13 cryogel with 0.8 wt.% MAETAC following 4 h of incubation provided cell adherence comparable to that previously reported for PEG-based hydrogels, e.g., non-activated [68,69] and RGD-modified ones [70], although these were incubated with the cells for 24–72 h. Higher concentrations of MAETAC clearly decreased the interaction of cells with OPF cryogels (Figure 4), presumably due to the inhibition of cell adhesion by excessively charged polymer chains, and these cryogel compositions led to changes in the material structure (Figure 2 and Figure 3). 

To induce specific cellular responses such as neuronal differentiation, the synthesized cryogels can be additionally modified with neuroactive components such as peptide factors. The feasibility of in situ functionalization of β-cyclodextrin-bearing cryogels with adamantylated ECM-derived peptides via host-guest interactions was previously demonstrated [30,71]. This immobilization strategy was proven to be effective for the controllable bulk modification of cryogel materials with synthetic peptide compositions, and it can be further exploited for the activation of OPF-based cryogels for SC repair applications. 

## 4. Materials and Methods

### 4.1. Materials

Fumaryl chloride (95%), triethylamine chloride (≥99%), poly(ethylene glycol) (PEG) (1500 Da), poly(ethylene glycol) diacrylate (PEGDA, *M_n_* = 575), N,N,N′,N′- tetramethylethylenediamine (TEMED, ≥99%), ammonium persulfate (APS), and dichloromethane (DCM) were purchased from Sigma-Aldrich (Saint Louis, MO, USA).

Cell culture media and reagents were purchased from Paneco (Moscow, Russia). The 3-(4,5-Dimethylthiazol-2-yl)-5-(3-carboxymethoxyphenyl)-2-(4-sulfophenyl)-2H-tetrazolium (MTS reagent) was purchased from Promega (Madison, WI, USA). PC-12 rat pheochromacytoma and SH-SY5Y human neuroblastoma cell lines were obtained from the American Type Culture Collection (Manassas, VA, USA). Cresyl violet acetate (Acros Organic) and 4,6-diamino-2-phenylindol (DAPI) (Sigma-Aldrich) were used for cell staining.

### 4.2. Synthesis and Characterization of OPF Macromer

OPF was synthesized as previously described [72]. Briefly, 50 g of PEG pre-dried in toluene by azeotropic distillation were dissolved in 320 mL of anhydrous dichloromethane (DCM). An amount of 30 mL of anhydrous fumaryl chloride (the fumaryl chloride:PEG molar ratio was 0.9:1) and 30 mL of triethylamine (the triethylamine:fumaryl chloride molar ratio was 2:1) was added dropwise from two funnels connected to a three-necked round-bottomed flask containing the PEG solution. The reaction was conducted under stirring and argon-purging conditions on ice. The reaction mixture was stirred at room temperature for 48 h, followed by DCM removal using a rotary evaporator (at 30 °C). The OPF product was then purified by crystallization in ethyl acetate, collected by filtration, washed using ethyl ether, and dried under a vacuum for 10 h.

The structure of the obtained OPF macromer was analyzed by ^1^H and ^13^C NMR spectroscopy after dissolution in deuterium oxide (D_2_O) (Figure S2). The spectra were recorded on a Bruker Avance-400 NMR spectrometer (Billerica, MA, USA) (400.0 MHz, ^1^H; 100.6 MHz, ^13^C).

### 4.3. Preparation of OPF-Based Cryogels

OPF-based cryogels were synthesized by vinyl addition polymerization in an aqueous solution using variable concentrations of constituents (Table 1). The reaction mixture was purged with N_2_, followed by the addition of 0.6 wt.% ammonium persulfate (APS) and 0.3 wt.% TEMED (as an initiator and activator of free radicals) upon stirring. The resultant solution was poured into transparent cylindrical glass tubes (1 cm inner diameter), placed in a cooling thermostat at a temperature of −12 °C for 4 h, and then transferred into a freezer (−18 °C, 24 h) to complete polymerization. The product was thawed at room temperature and washed with deionized water to obtain a cylindrical cryogel.

To prepare non-porous hydrogels, the mixture of constituents with APS and TEMED was kept at room temperature until a stable gel was formed.

### 4.4. Fourier Transform Infrared (FTIR) Spectroscopy

Prior to the analysis, the OPF-based cryogels were washed with milli-Q grade water and freeze-dried by lyophilization. The attenuated total reflectance (ATR) FTIR spectra of the lyophilized cryogels as well as constituents (PEG, MAETAC, and OPF) were recorded on a Frontier spectrometer (PerkinElmer, Waltham, MA, USA) in the wavenumber range 4000–400 cm^–1^ with a resolution of 1 cm^–1^.

### 4.5. Analysis of Swelling and Viscoelastic Properties of The Cryogels

The cryogel samples were cut into round, 3 mm-thick disks and equilibrated with deionized water. The swelling ratio (SR) was calculated according to the formula: SR = (m_x_ − m_0_)/m_0_ × 100%, where m_x(1, 2)_ represents the mass of fully hydrated cryogels (m_1_) or partially hydrated cryogels (m_2_) after the removal of weakly bound water (or capillary water, CW) [30]. The mass of completely dried cryogels (m_0_) was recorded after the removal of polymer-bound water (PW) from the materials and was placed in a heating thermostat at 90 °C. The volume fraction of CW relative to the total water volume of the swollen cryogels was calculated with the following formula: V_CW_ (%) = (m_1_ − m_2_)/(m_1_ − m_0_) × 100%.

The rheological properties of the swollen cryogels were analyzed using an MCR 302 rotational rheometer (Anton Paar, Ashland, VA, USA) at 25 °C. The strain sweep and frequency sweep tests were performed by applying a 0.01–100% strain (ω = 10 rad s^–1^) and 0.01–100 rad s^–1^ angular frequencies (γ = 0.5 %), respectively. The frequency dependencies of the storage (G′) and loss (G″) moduli of the materials were recorded within a linear viscoelastic region (LVR).

The porous structure of the cryogels was analyzed using laser scanning confocal microscopy (LSCM) with a LSM 780 microscope (Carl Zeiss) equipped with argon laser excitation (488 nm). Zeiss ZEN black software was used for acquisition.

### 4.6. Cell Maintenance and Seeding

PC-12 rat pheochromacytoma and SH-SY5Y human neuroblastoma cells were maintained in sterile DMEM supplemented with 10% horse serum and 5% fetal bovine serum (FBS) (PC-12) or 10% FBS (SH-SY5Y). Penicillin (100 U/mL), streptomycin (100 μg/mL), and L-glutamine (2 mM) were added to the culture medium. The cells were cultured under standard conditions in a temperature- and humidity-controlled incubator (37 ℃ and 5% CO_2_). The culture medium was refreshed every 2 days. 

Prior to cell seeding, the gels were treated with penicillin (2.5 kU/mL) and streptomycin (2.5 mg/mL) solutions for 1 h, rinsed with Hanks’ Balanced Salt Solution (HBSS), and equilibrated in the culture medium. The cells were seeded onto the cryogel surface using the top seeding method [37] in a 12-well plate at a density of 6.4 × 10^4^ cells/cm^2^ of the cryogel area and incubated for 1.5 h under standard culture conditions to allow for cell attachment.

### 4.7. Study of Cell Migration

Cryogel samples were cut into cylindrical pieces of 10 mm in height and placed vertically in a 12-well plate. A complete cell culture medium with 10% FBS was added to the wells to a level of 5 mm to cover half of the material height. An aliquot of the cell suspension in serum-free DMEM (6.4 × 10^4^ cells/cm^2^) was spread on the cryogel surface and cultured for 6 h under standard conditions. The cryogels were then washed with PBS and incubated in 4% *p*-formaldehyde in PBS for 2 h. The fixed cells were stained with DAPI nuclear dye and visualized by LSCM on a LSM 780 microscope (Carl Zeiss).

### 4.8. Cell Adherence Assay

PC-12 and SH-SY5Y cells were seeded on top of the cryogels, pre-incubated in serum-free medium at a cell density of 6.4 × 10^4^ cells/cm^2^, and then allowed to adhere for 4 h. Unadhered cells were collected by washing them with HBSS and counting them using a hemocytometer. Cell adherence efficiency was determined according to the following formula:

*cell adherence* (*%*) = (*n_i_* − *n_ii_*)/*n_i_* × 100 (1)

where *n_i_* represents the number of initially seeded cells, while *n_ii_* is the number of unattached cells [71].

### 4.9. Cell Detection in the Cryogels

PC-12 and SH-SY5Y cells were seeded on top of the cryogels, pre-incubated in full medium at a cell density of 6.4 × 10^4^ cells/cm^2^, and cultured for 72 h under standard conditions. The cryogels with grown cells were transferred into new wells containing fresh culture medium supplemented with MTS/PMS reagents to assess the cell metabolic activity [38,73]. After incubating the samples for 1.5 h in a CO_2_-incubator (37 °C, 5% CO_2_), the optical absorbance of the generated formazan product was detected at 490 nm on an Infinite M200 PRO microplate analyzer (Tecan) as a measure of a viable cell number.

For the bright-field microscopy analysis, the cryogels with grown cells were fixed with 4% *p*-formaldehyde. After a gentle wash with PBS, the fixed cells were stained with cresyl violet (0.1% *w*/*v* in deionized water) for 5 min and visualized using an AxioObserver Z1 microscope (Carl Zeiss).

### 4.10. Statistical Analysis 

Data were presented as mean ± SD. The statistical significance was determined when appropriate with a one-way analysis of variance (ANOVA), followed by Tukey’s multiple comparison post-test or with Student’s *t*-test analysis (* *p* < 0.05, ** *p* < 0.01, and *** *p* < 0.001).

## Figures and Tables

**Table 1:** Mass content of constituents (wt.%) used to synthesize OPF-based cryogels and images of the obtained cryogels.

	Gel	OPF	PEGDA	MAETAC	Images
**Group A**	OPF-1	0.5	0	0	NA
	OPF-2	1	0	0	NA
	OPF-3	2	0	0	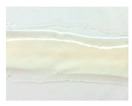
	OPF-4	4	0	0	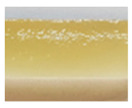
	OPF-5	6	0	0	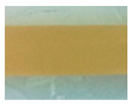
**Group B**	OPF-6	4	0.32	0	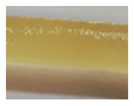
	OPF-7	4	0.8	0	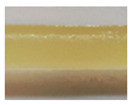
	OPF-8	4	2	0	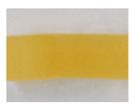
	OPF-9	4	3	0	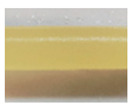
	OPF-10	4	4	0	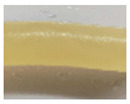
**Group C**	OPF-11	4	2	0.32	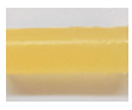
	OPF-12	4	2	0.506	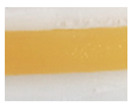
	OPF-13	4	2	0.8	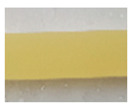
	OPF-14	4	2	1.265	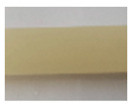
	OPF-15	4	2	2	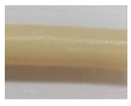
**Group D**	OPF-16	4	0	0.8	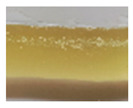
	OPF-17	4	0.32	0.8	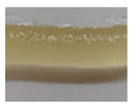
	OPF-18	4	0.8	0.8	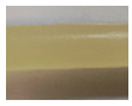
	OPF-19	4	3	0.8	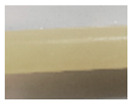

**Table 2:** Mean values of the storage modulus (G′), the loss modulus (G″), and the G′/G″ ratio of OPF-based cryogels recorded at ω = 1 rad s^−1^. Cryogel compositions (wt. %) are shown in parentheses. The data are presented as mean ± SD.

	Gel	G′ (Pa)	G″ (Pa)	G′/G″
**Group A** (*OPF x%*)	**OPF-3** (OPF 2%)	41 ± 15	1.2 ± 0.5	34 ± 2
	**OPF-4** (OPF 4%)	239 ± 66	6 ± 2	39 ± 3
**Group B** (OPF 4%, *PEGDA y%*)	**OPF-6** (PEGDA 0.32%)	937 ± 117	15.8 ± 0.1	59 ± 7
	**OPF-7** (PEGDA 0.8%)	1800 ± 42	32 ± 9	58 ± 16
	**OPF-8** (PEGDA 2%)	2730 ± 353	48.0 ± 0.2	57 ± 7
	**OPF-9** (PEGDA 3%)	4910 ± 608	139 ± 1	35 ± 4
	**OPF-10** (PEGDA 4%)	9500 ± 579	1275 ± 63	7.4 ± 0.1
**Group C** (OPF 4%, PEGDA 2%, *MAETAC z%*)	**OPF-11** (MAETAC 0.32%)	4445 ± 388	122 ± 38	38 ± 9
	**OPF-12** (MAETAC 0.51%)	5045 ± 219	122 ± 8	42 ± 1
	**OPF-13** (MAETAC 0.8%)	5595 ± 5	144 ± 16	39 ± 4
	**OPF-14** (MAETAC 1.27%)	2720 ± 495	102 ± 33	27 ± 4
	**OPF-15** (MAETAC 2%)	2610 ± 70	98 ± 20	27 ± 6
**Group D** (OPF 4 %, *PEGDA y%*, MAETAC 0.8%)	**OPF-16** (PEGDA 0%)	98 ± 4	12 ± 1	8.5 ± 0.2
	**OPF-17** (PEGDA 0.32%)	255 ± 1	18 ± 4	15 ± 3
	**OPF-18** (PEGDA 0.8%)	2345 ± 35	56 ± 5	42 ± 3
	**OPF-19** (PEGDA 3%)	5875 ± 685	158 ± 11	37 ± 2

**Table 3:** Reported values of storage modulus (G′) and loss modulus (G″) for gel-based materials used for SC repair.

Gel	G′ (Pa)	G″ (Pa)	Ref
Hyaluronic acid-methylcellulose hydrogels with embedded BDNF-loaded PLGA microparticles	2200–4200	320–540	[54]
RGD peptide-modified poly[*N*-(2-hydroxypropyl)methacrylamide] (PHPMA) hydrogel	100	~10	[59]
Gelled ECM components (e.g., collagen and GAG) extracted from decellularized sciatic nerves	171	25	[49]
Gelled ECM components (e.g., collagen and GAG) extracted from decellularized spinal cord	139–775	16–94	[51]
Collagen-laminin-hyaluronic acid ECM-mimicking hydrogel	43	7	[50]
Dopamine-modified chitosan hydrogels	1000–1500	20–40	[55]
P_11_-8 peptide-enriched PCL fibers integrated with glycidylmethacrylated collagen hydrogels	340–2574	61–264	[23]
Lauryl-VVAG nanofiber-forming sequence conjugated with neuroactive peptide motifs	1000–2020	150–700	[64]
Electroconductive polypyrrole-modified chondroitin sulphate-gelatin hydrogels	423–1600	40–600	[52]
Graphene-crosslinked collagen cryogel	1200	110	[57]
Polysaccharide-based composite hydrogels composed of Ca-alginate	26–6040	6–3030	[53]
FGF-loaded methacrylate-silk fibroin hydrogels	300–2000	10–30	[58]

## Data Availability

The data presented in this study are contained within the article and Supplementary Materials.

## Supplementary Materials

The following supporting information can be downloaded at: https://www.mdpi.com/article/10.3390/gels9020105/s1, Figure S1: FTIR spectra of PEG, OPF, MAETAC (A), and lyophilized OPF/PEGDA cryogels with an increased MAETAC amount (B). The relationship between the MAETAC concentration in the reaction solution and the FTIR signal of MAETAC in cryogels determined by the peak intensity ratio 950/1100 cm^−1^ (C); Figure S2: ^1^H NMR spectrum (400 MHz, D_2_O, ambient temperature) (A) and ^13^C–{^1^H} NMR spectrum (100.6 MHz, D_2_O, ambient temperature) (B) of oligo (poly (ethylene glycol) fumarate) (OPF) macromer; Figure S3: Relationships between G′/G″ moduli of OPF cryogels (OPF 4 wt.%) and the amount of PEGDA (A) and OPF/PEGDA cryogels (OPF 4 wt.%, PEGDA 2 wt.%) and the amount of MAETAC (B); Figure S4: Overall swelling index (total water content %) for OPF-based cryogels; Figure S5: Frequency sweep analysis of OPF-based cryogels (group D). The measurement of frequency dependence of storage (G′) and loss (G″) moduli was performed within LVR at δ = 0.5% strain deformation. Cryogel compositions (wt. %) are shown in parentheses.

## References

[1] Siddiqui A.M., Islam R., Cuellar C.A., Silvernail J.L., Knudsen B., Curley D.E., Strickland T., Manske E., Suwan P.T., Latypov T. (2021). Newly Regenerated Axons via Scaffolds Promote Sub-Lesional Reorganization and Motor Recovery with Epidural Electrical Stimulation. npj Regen. Med..

[2] Alizadeh A., Dyck S.M., Karimi-Abdolrezaee S. (2019). Traumatic Spinal Cord Injury: An Overview of Pathophysiology, Models and Acute Injury Mechanisms. Front. Neurol..

[3] Khorasanizadeh M.H., Yousefifard M., Eskian M., Lu Y., Chalangari M., Harrop J.S., Jazayeri S.B., Seyedpour S., Khodaei B., Hosseini M. (2019). Neurological Recovery Following Traumatic Spinal Cord Injury: A Systematic Review and Meta-Analysis. J. Neurosurg. Spine.

[4] Zhang Q., Shi B., Ding J., Yan L., Thawani J.P., Fu C., Chen X. (2019). Polymer Scaffolds Facilitate Spinal Cord Injury Repair. Acta Biomater..

[5] Shrestha B., Coykendall K., Li Y., Moon A., Priyadarshani P., Yao L. (2014). Repair of Injured Spinal Cord Using Biomaterial Scaffolds and Stem Cells. Stem Cell Res. Ther..

[6] Hassannejad Z., Zadegan S.A., Vaccaro A.R., Rahimi-Movaghar V., Sabzevari O. (2019). Biofunctionalized Peptide-Based Hydrogel as an Injectable Scaffold for BDNF Delivery Can Improve Regeneration after Spinal Cord Injury. Injury.

[7] Liu S., Xie Y.Y., Wang B. (2019). Role and Prospects of Regenerative Biomaterials in the Repair of Spinal Cord Injury. Neural Regen. Res..

[8] Ghane N., Beigi M.H., Labbaf S., Nasr-Esfahani M.H., Kiani A. (2020). Design of Hydrogel-Based Scaffolds for the Treatment of Spinal Cord Injuries. J. Mater. Chem. B.

[9] Qu W., Chen B., Shu W., Tian H., Ou X., Zhang X., Wang Y., Wu M. (2020). Polymer-Based Scaffold Strategies for Spinal Cord Repair and Regeneration. Front. Bioeng. Biotechnol..

[10] Yang Y., Fan Y., Zhang H., Zhang Q., Zhao Y., Xiao Z., Liu W., Chen B., Gao L., Sun Z. (2021). Small Molecules Combined with Collagen Hydrogel Direct Neurogenesis and Migration of Neural Stem Cells after Spinal Cord Injury. Biomaterials.

[11] Yao M., Li J., Zhang J., Ma S., Wang L., Gao F., Guan F. (2021). Dual-Enzymatically Cross-Linked Gelatin Hydrogel Enhances Neural Differentiation of Human Umbilical Cord Mesenchymal Stem Cells and Functional Recovery in Experimental Murine Spinal Cord Injury. J. Mater. Chem. B.

[12] Zarei-Kheirabadi M., Sadrosadat H., Mohammadshirazi A., Jaberi R., Sorouri F., Khayyatan F., Kiani S. (2020). Human Embryonic Stem Cell-Derived Neural Stem Cells Encapsulated in Hyaluronic Acid Promotes Regeneration in a Contusion Spinal Cord Injured Rat. Int. J. Biol. Macromol..

[13] Yu Z., Li H., Xia P., Kong W., Chang Y., Fu C., Wang K., Yang X., Qi Z. (2020). Application of Fibrin-Based Hydrogels for Nerve Protection and Regeneration after Spinal Cord Injury. J. Biol. Eng..

[14] Grijalvo S., Nieto-Díaz M., Maza R.M., Eritja R., Díaz D.D. (2019). Alginate Hydrogels as Scaffolds and Delivery Systems to Repair the Damaged Spinal Cord. Biotechnol. J..

[15] Kong X.B., Tang Q.Y., Chen X.Y., Tu Y., Sun S.Z., Sun Z.L. (2017). Polyethylene Glycol as a Promising Synthetic Material for Repair of Spinal Cord Injury. Neural Regen. Res..

[16] Lu X., Perera T.H., Aria A.B., Callahan L.A.S. (2018). Polyethylene Glycol in Spinal Cord Injury Repair: A Critical Review. J. Exp. Pharmacol..

[17] Hejcl A., Urdzikova L., Sedy J., Lesny P., Pradny M., Michalek J., Burian M., Hajek M., Zamecnik J., Jendelova P. (2008). Acute and Delayed Implantation of Positively Charged 2-Hydroxyethyl Methacrylate Scaffolds in Spinal Cord Injury in the Rat: Laboratory Investigation. J. Neurosurg. Spine.

[18] Sun F., Shi T., Zhou T., Dong D., Xie J., Wang R., An X., Chen M., Cai J. (2017). 3D Poly(Lactic-Co-Glycolic Acid) Scaffolds for Treating Spinal Cord Injury. J. Biomed. Nanotechnol..

[19] Donoghue P.S., Lamond R., Boomkamp S.D., Sun T., Gadegaard N., Riehle M.O., Barnett S.C. (2013). The Development of a É-Polycaprolactone Scaffold for Central Nervous System Repair. Tissue Eng. Part A.

[20] Perale G., Rossi F., Sundstrom E., Bacchiega S., Masi M., Forloni G., Veglianese P. (2011). Hydrogels in Spinal Cord Injury Repair Strategies. ACS Chem. Neurosci..

[21] Kim K.D., Lee K.S., Coric D., Chang J.J., Harrop J.S., Theodore N., Toselli R.M. (2021). A Study of Probable Benefit of a Bioresorbable Polymer Scaffold for Safety and Neurological Recovery in Patients with Complete Thoracic Spinal Cord Injury: 6-Month Results from the INSPIRE Study. J. Neurosurg. Spine.

[22] Xiao Z., Tang F., Zhao Y., Han G., Yin N., Li X., Chen B., Han S., Jiang X., Yun C. (2018). Significant Improvement of Acute Complete Spinal Cord Injury Patients Diagnosed by a Combined Criteria Implanted with NeuroRegen Scaffolds and Mesenchymal Stem Cells. Cell Transplant..

[23] Golland B., Tipper J.L., Hall R.M., Tronci G., Russell S.J. (2022). A Biomimetic Nonwoven-Reinforced Hydrogel for Spinal Cord Injury Repair. Polymers.

[24] Wang Y., Lv H.Q., Chao X., Xu W.X., Liu Y., Ling G.X., Zhang P. (2022). Multimodal Therapy Strategies Based on Hydrogels for the Repair of Spinal Cord Injury. Mil. Med. Res..

[25] Hakim J.S., Esmaeili Rad M., Grahn P.J., Chen B.K., Knight A.M., Schmeichel A.M., Isaq N.A., Dadsetan M., Yaszemski M.J., Windebank A.J. (2015). Positively Charged Oligo [Poly (Ethylene Glycol) Fumarate] Scaffold Implantation Results in a Permissive Lesion Environment after Spinal Cord Injury in Rat. Tissue Eng. Part A.

[26] Chen B.K., Madigan N.N., Hakim J.S., Dadsetan M., McMahon S.S., Yaszemski M.J., Windebank A.J. (2018). GDNF Schwann Cells in Hydrogel Scaffolds Promote Regional Axon Regeneration, Remyelination and Functional Improvement after Spinal Cord Transection in Rats. J. Tissue Eng. Regen. Med..

[27] Siddiqui A.M., Oswald D., Papamichalopoulos S., Kelly D., Summer P., Polzin M., Hakim J., Schmeichel A.M., Chen B., Yaszemski M.J. (2021). Defining Spatial Relationships between Spinal Cord Axons and Blood Vessels in Hydrogel Scaffolds. Tissue Eng. Part A.

[28] Eigel D., Werner C., Newland B. (2021). Cryogel Biomaterials for Neuroscience Applications. Neurochem. Int..

[29] Savina I.N., Zoughaib M., Yergeshov A.A. (2021). Design and Assessment of Biodegradable Macroporous Cryogels as Advanced Tissue Engineering and Drug Carrying Materials. Gels.

[30] Luong T.D., Zoughaib M., Garifullin R., Kuznetsova S., Guler M.O., Abdullin T.I. (2020). In Situ Functionalization of Poly(Hydroxyethyl Methacrylate) Cryogels with Oligopeptides via β-Cyclodextrin-Adamantane Complexation for Studying Cell-Instructive Peptide Environment. ACS Appl. Bio Mater..

[31] Silva D., Sousa R.A., Salgado A.J. (2021). Hydrogels as Delivery Systems for Spinal Cord Injury Regeneration. Mater. Today Bio.

[32] Jo S., Shin H., Shung A.K., Fisher J.P., Mikos A.G. (2001). Synthesis and Characterization of Oligo (Poly (Ethylene Glycol) Fumarate) Macromer. Macromolecules.

[33] Park H., Guo X., Temenoff J.S., Tabata Y., Caplan A.I., Kasper F.K., Mikos A.G. (2009). Effect of Swelling Ratio of Injectable Hydrogel Composites on Chondrogenic Differentiation of Encapsulated Rabbit Marrow Mesenchymal Stem Cells in Vitro. Biomacromolecules.

[34] Li W., Huang A., Zhong Y., Huang L., Yang J., Zhou C., Zhou L., Zhang Y., Fu G. (2020). Laminin-Modified Gellan Gum Hydrogels Loaded with the Nerve Growth Factor to Enhance the Proliferation and Differentiation of Neuronal Stem Cells. RSC Adv..

[35] Luong D., Yergeshov A.A., Zoughaib M., Sadykova F.R., Gareev B.I., Savina I.N., Abdullin T.I. (2019). Transition Metal-Doped Cryogels as Bioactive Materials for Wound Healing Applications. Mater. Sci. Eng. C.

[36] Petrov P., Petrova E., Tchorbanov B., Tsvetanov C.B. (2007). Synthesis of Biodegradable Hydroxyethylcellulose Cryogels by UV Irradiation. Polymer.

[37] Labour M.N., Banc A., Tourrette A., Cunin F., Verdier J.M., Devoisselle J.M., Marcilhac A., Belamie E. (2012). Thick Collagen-Based 3D Matrices Including Growth Factors to Induce Neurite Outgrowth. Acta Biomater..

[38] Zoughaib M.H., Luong D.T., Siraeva Z.Y., Yergeshov A.A., Salikhova T.I., Kuznetsova S.V., Kiyamova R.G., Abdullin T.I. (2019). Tumor Cell Behavior in Porous Hydrogels: Effect of Application Technique and Doxorubicin Treatment. Bull. Exp. Biol. Med..

[39] Jurga M., Dainiak M.B., Sarnowska A., Jablonska A., Tripathi A., Plieva F.M., Savina I.N., Strojek L., Jungvid H., Kumar A. (2011). The Performance of Laminin-Containing Cryogel Scaffolds in Neural Tissue Regeneration. Biomaterials.

[40] Mahumane G.D., Kumar P., Du Toit L.C., Choonara Y.E., Pillay V. (2018). 3D Scaffolds for Brain Tissue Regeneration: Architectural Challenges. Biomater. Sci..

[41] Lam J., Kim K., Lu S., Tabata Y., Scott D.W., Mikos A.G., Kurtis Kasper F. (2014). A Factorial Analysis of the Combined Effects of Hydrogel Fabrication Parameters on the in Vitro Swelling and Degradation of Oligo (Poly (Ethylene Glycol) Fumarate) Hydrogels. J. Biomed. Mater. Res. Part A.

[42] Chen B.K., Knight A.M., Madigan N.N., Gross L.A., Dadsetan M., Nesbitt J.J., Rooney G.E., Currier B.L., Yaszemski M.J., Spinner R.J. (2011). Comparison of Polymer Scaffolds in Rat Spinal Cord: A Step toward Quantitative Assessment of Combinatorial Approaches to Spinal Cord Repair. Biomaterials.

[43] Georges P.C., Miller W.J., Meaney D.F., Sawyer E.S., Janmey P.A. (2006). Matrices with Compliance Comparable to That of Brain Tissue Select Neuronal over Glial Growth in Mixed Cortical Cultures. Biophys. J..

[44] Levental I., Georges P.C., Janmey P.A. (2007). Soft Biological Materials and Their Impact on Cell Function. Soft Matter.

[45] Gefen A., Gefen N., Zhu Q., Raghupathi R., Margulies S.S. (2003). Age-Dependent Changes in Material Properties of the Brain and Braincase of the Rat. J. Neurotrauma.

[46] Cheng S., Clarke E.C., Bilston L.E. (2008). Rheological Properties of the Tissues of the Central Nervous System: A Review. Med. Eng. Phys..

[47] Ramo N.L., Troyer K.L., Puttlitz C.M. (2018). Viscoelasticity of Spinal Cord and Meningeal Tissues. Acta Biomater..

[48] Woerly S., Pinet E., de Robertis L., van Diep D., Bousmina M. (2001). Spinal Cord Repair with PHPMA Hydrogel Containing RGD Peptides (NeuroGel^TM^). Biomaterials.

[49] Cornelison R.C., Gonzalez-Rothi E.J., Porvasnik S.L., Wellman S.M., Park J.H., Fuller D.D., Schmidt C.E. (2018). Injectable Hydrogels of Optimized Acellular Nerve for Injection in the Injured Spinal Cord. Biomed. Mater..

[50] Geissler S.A., Sabin A.L., Besser R.R., Gooden O.M., Shirk B.D., Nguyen Q.M., Khaing Z.Z., Schmidt C.E. (2018). Biomimetic Hydrogels Direct Spinal Progenitor Cell Differentiation and Promote Functional Recovery after Spinal Cord Injury. J. Neural Eng..

[51] Medberry C.J., Crapo P.M., Siu B.F., Carruthers C.A., Wolf M.T., Nagarkar S.P., Agrawal V., Jones K.E., Kelly J., Johnson S.A. (2013). Hydrogels Derived from Central Nervous System Extracellular Matrix. Biomaterials.

[52] Luo Y., Fan L., Liu C., Wen H., Wang S., Guan P., Chen D., Ning C., Zhou L., Tan G. (2022). An Injectable, Self-Healing, Electroconductive Extracellular Matrix-Based Hydrogel for Enhancing Tissue Repair after Traumatic Spinal Cord Injury. Bioact. Mater..

[53] Des Rieux A., de Berdt P., Ansorena E., Ucakar B., Damien J., Schakman O., Audouard E., Bouzin C., Auhl D., Simõn-Yarza T. (2014). Vascular Endothelial Growth Factor-Loaded Injectable Hydrogel Enhances Plasticity in the Injured Spinal Cord. J. Biomed. Mater. Res. Part A.

[54] Khaing Z.Z., Agrawal N.K., Park J.H., Xin S., Plumton G.C., Lee K.H., Huang Y.J., Niemerski A.L., Schmidt C.E., Grau J.W. (2016). Localized and Sustained Release of Brain-Derived Neurotrophic Factor from Injectable Hydrogel/Microparticle Composites Fosters Spinal Learning after Spinal Cord Injury. J. Mater. Chem. B.

[55] Liu K., Dong X., Wang Y., Wu X., Dai H. (2022). Dopamine-Modified Chitosan Hydrogel for Spinal Cord Injury. Carbohydr. Polym..

[56] Huang F., Chen T., Chang J., Zhang C., Liao F., Wu L., Wang W., Yin Z. (2021). A Conductive Dual-Network Hydrogel Composed of Oxidized Dextran and Hyaluronic-Hydrazide as BDNF Delivery Systems for Potential Spinal Cord Injury Repair. Int. J. Biol. Macromol..

[57] Agarwal G., Kumar N., Srivastava A. (2021). Highly Elastic, Electroconductive, Immunomodulatory Graphene Crosslinked Collagen Cryogel for Spinal Cord Regeneration. Mater. Sci. Eng. C.

[58] Zhou L., Wang Z., Chen D., Lin J., Li W., Guo S., Wu R., Zhao X., Lin T., Chen G. (2022). An Injectable and Photocurable Methacrylate-Silk Fibroin Hydrogel Loaded with BFGF for Spinal Cord Regeneration. Mater. Des..

[59] George J., Hsu C.C., Nguyen L.T.B., Ye H., Cui Z. (2020). Neural Tissue Engineering with Structured Hydrogels in CNS Models and Therapies. Biotechnol. Adv..

[60] Banerjee A., Arha M., Choudhary S., Ashton R.S., Bhatia S.R., Schaffer D.V., Kane R.S. (2009). The Influence of Hydrogel Modulus on the Proliferation and Differentiation of Encapsulated Neural Stem Cells. Biomaterials.

[61] Saha K., Keung A.J., Irwin E.F., Li Y., Little L., Schaffer D.V., Healy K.E. (2008). Substrate Modulus Directs Neural Stem Cell Behavior. Biophys. J..

[62] Hynes S.R., Rauch M.F., Bertram J.P., Lavik E.B. (2009). A Library of Tunable Poly (Ethylene Glycol)/Poly(L-Lysine) Hydrogels to Investigate the Material Cues That Influence Neural Stem Cell Differentiation. J. Biomed. Mater. Res. Part A.

[63] Kubinová Š., Horák D., Hejčl A., Plichta Z., Kotek J., Proks V., Forostyak S., Syková E. (2015). SIKVAV-Modified Highly Superporous PHEMA Scaffolds with Oriented Pores for Spinal Cord Injury Repair. J. Tissue Eng. Regen. Med..

[64] Sever-Bahcekapili M., Yilmaz C., Demirel A., Kilinc M.C., Dogan I., Caglar Y.S., Guler M.O., Tekinay A.B. (2021). Neuroactive Peptide Nanofibers for Regeneration of Spinal Cord after Injury. Macromol. Biosci..

[65] Shahriari D., Koffler J.Y., Tuszynski M.H., Campana W.M., Sakamoto J.S. (2017). Hierarchically Ordered Porous and High-Volume Polycaprolactone Microchannel Scaffolds Enhanced Axon Growth in Transected Spinal Cords. Tissue Eng. Part A.

[66] Shahriari D., Koffler J., Lynam D.A., Tuszynski M.H., Sakamoto J.S. (2016). Characterizing the Degradation of Alginate Hydrogel for Use in Multilumen Scaffolds for Spinal Cord Repair. J. Biomed. Mater. Res. Part A.

[67] Thomas A.M., Kubilius M.B., Holland S.J., Seidlits S.K., Boehler R.M., Anderson A.J., Cummings B.J., Shea L.D. (2013). Channel Density and Porosity of Degradable Bridging Scaffolds on Axon Growth after Spinal Injury. Biomaterials.

[68] Fan C., Zhang C., Jing Y., Liao L., Liu L. (2013). Preparation and Characterization of a Biodegradable Hydrogel Containing Oligo(2,2-Dimethyltrimethylene Carbonate) Moieties with Tunable Properties. RSC Adv..

[69] Yu H., Chen X., Cai J., Ye D., Wu Y., Fan L., Liu P. (2019). Novel Porous Three-Dimensional Nanofibrous Scaffolds for Accelerating Wound Healing. Chem. Eng. J..

[70] Della Sala F., Biondi M., Guarnieri D., Borzacchiello A., Ambrosio L., Mayol L. (2020). Mechanical Behavior of Bioactive Poly (Ethylene Glycol) Diacrylate Matrices for Biomedical Application. J. Mech. Behav. Biomed. Mater..

[71] Zoughaib M., Luong D., Garifullin R., Gatina D.Z., Fedosimova S.V., Abdullin T.I. (2021). Enhanced Angiogenic Effects of RGD, GHK Peptides and Copper (II) Compositions in Synthetic Cryogel ECM Model. Mater. Sci. Eng. C.

[72] Kinard L.A., Kasper F.K., Mikos A.G. (2012). Synthesis of Oligo (Poly (Ethylene Glycol) Fumarate). Nat. Protoc..

[73] Yergeshov A.A., Zoughaib M., Ishkaeva R.A., Savina I.N., Abdullin T.I. (2022). Regenerative Activities of ROS-Modulating Trace Metals in Subcutaneously Implanted Biodegradable Cryogel. Gels.

