# Supplementary material for: Oligo (Poly (Ethylene Glycol) Fumarate)-Based Multicomponent Cryogels for Neural Tissue Replacement
Source: Gels. 2023 Jan 25;9(2):105. doi: 10.3390/gels9020105 (PMC9957547; doi:10.3390/gels9020105)
Supplement: Supplementary file 1 [file gels-09-00105-s001.zip › gels-2179207-supplementary.pdf]

Supplementary Materials

A

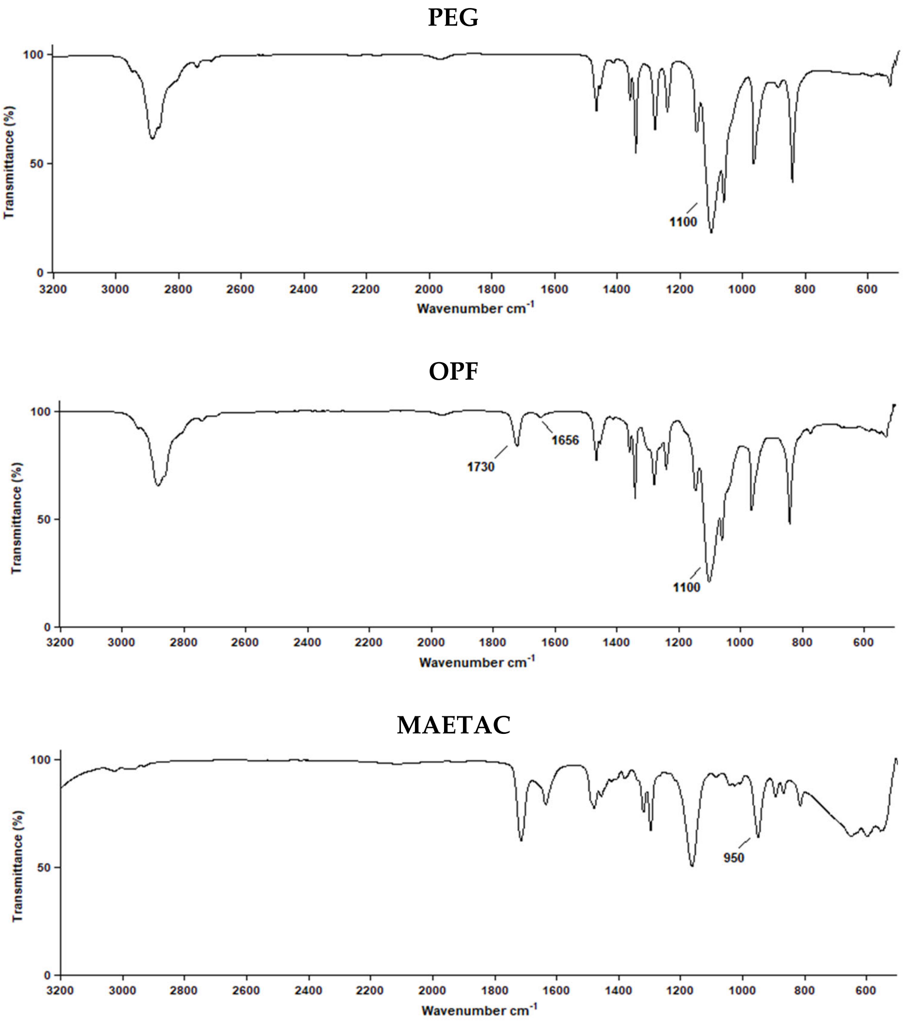

B

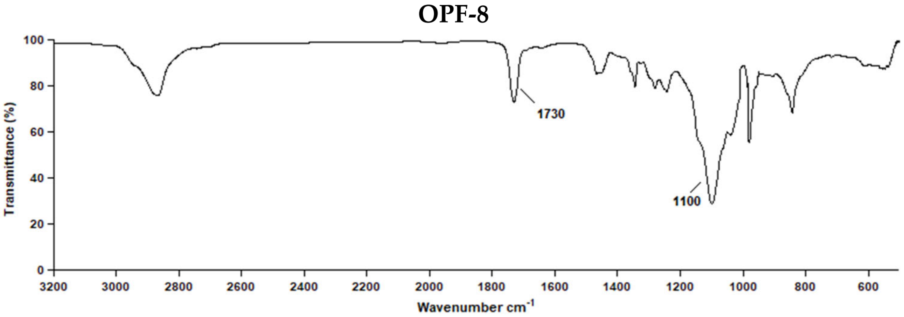

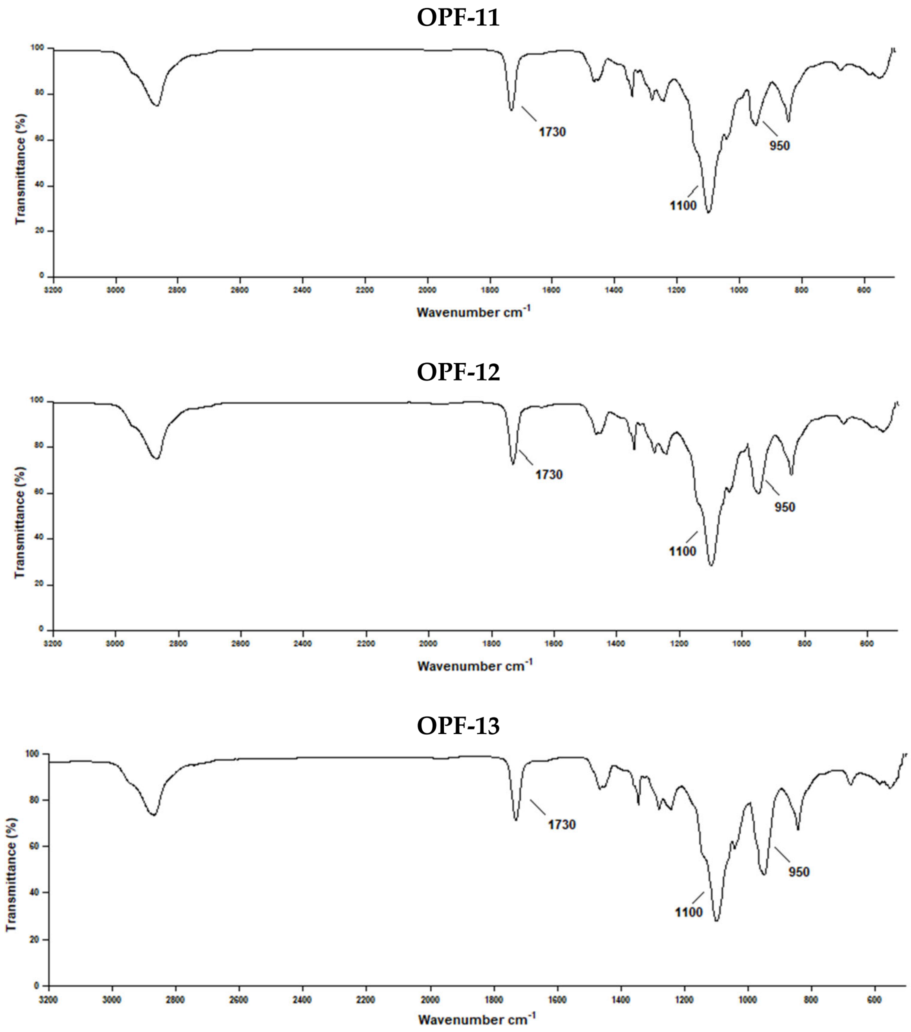

C

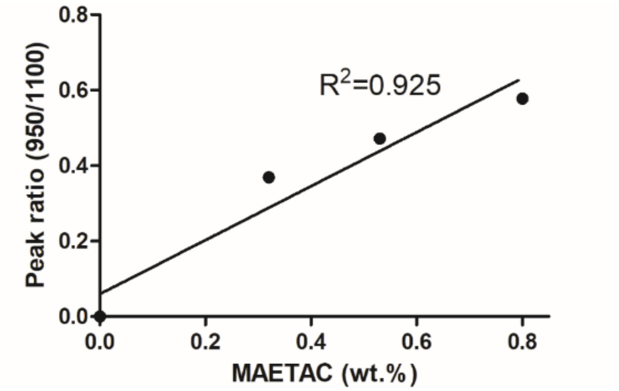

Figure S1. FTIR spectra of PEG, OPF, MAETAC (**A**) and lyophilized OPF/PEGDA cryogels with increased MAETAC amount (**B**). Relationship between MAETAC concentration in reaction solution and FTIR signal of MAETAC in cryogels determined by the peak intensity ratio 950/1100 cm^{-1} (**C**).

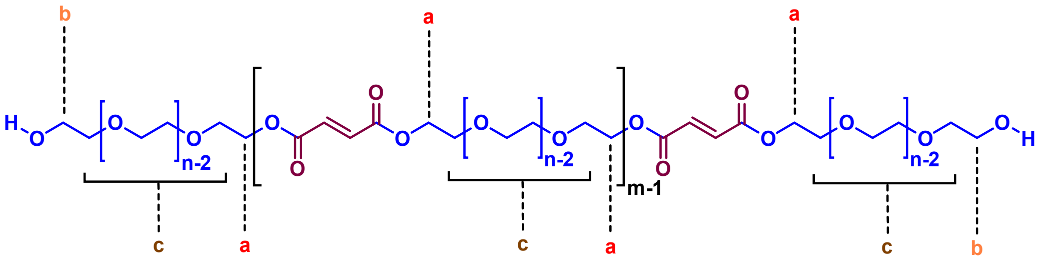

b
a
a
H
O
n-2
O
m-1
OH
c
b

A

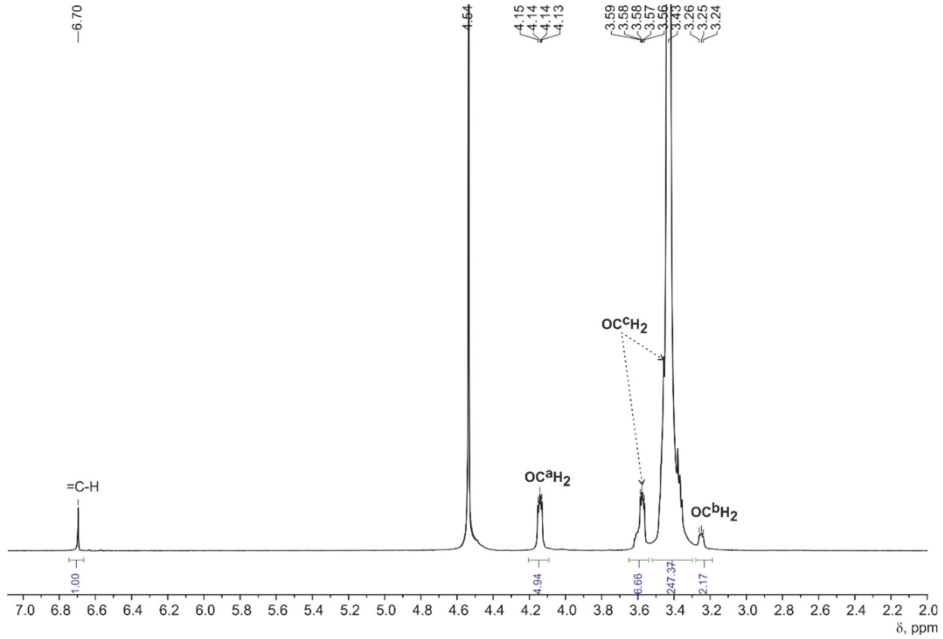

6.70
4.54
4.15
4.14
4.14
4.13
3.59
3.58
3.58
3.57
3.56
3.43
3.26
3.25
3.24
=C-H
$OC^{a}H_2$
$OC^{c}H_2$
$OC^{b}H_2$
1.00
4.94
6.66
247.37
2.17
δ, ppm

B

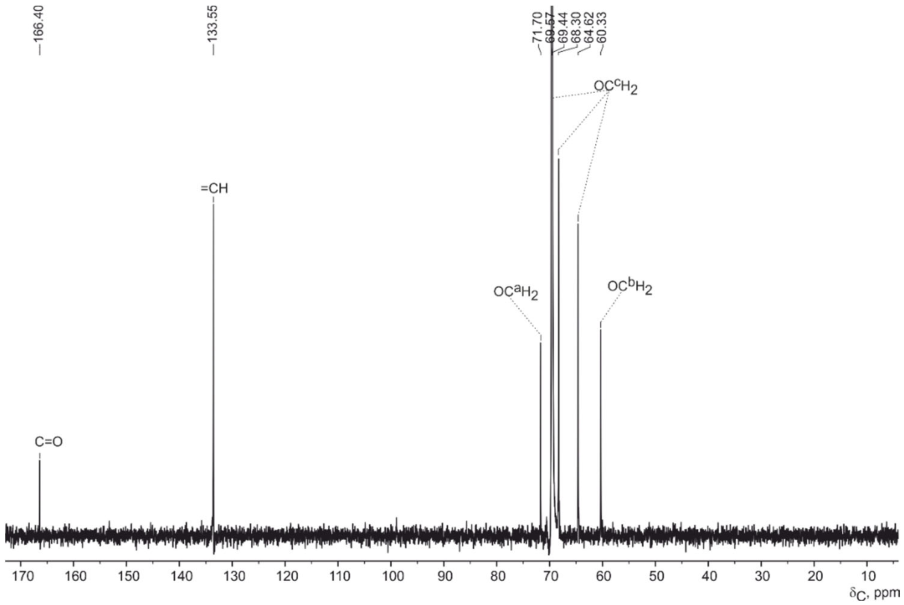

166.40
133.55
71.70
69.57
69.44
68.30
64.62
60.33
$OC^{c}H_2$
=CH
$OC^{a}H_2$
$OC^{b}H_2$
C=O
δ_C, ppm

Figure S2. ^{1}H NMR spectrum (400 MHz, D_2O, ambient temperature) (**A**) and ^{13}C–{^{1}H} NMR spectrum (100.6 MHz, D_2O, ambient temperature) (**B**) of oligo (poly(ethylene glycol) fumarate) (OPF) macromer.

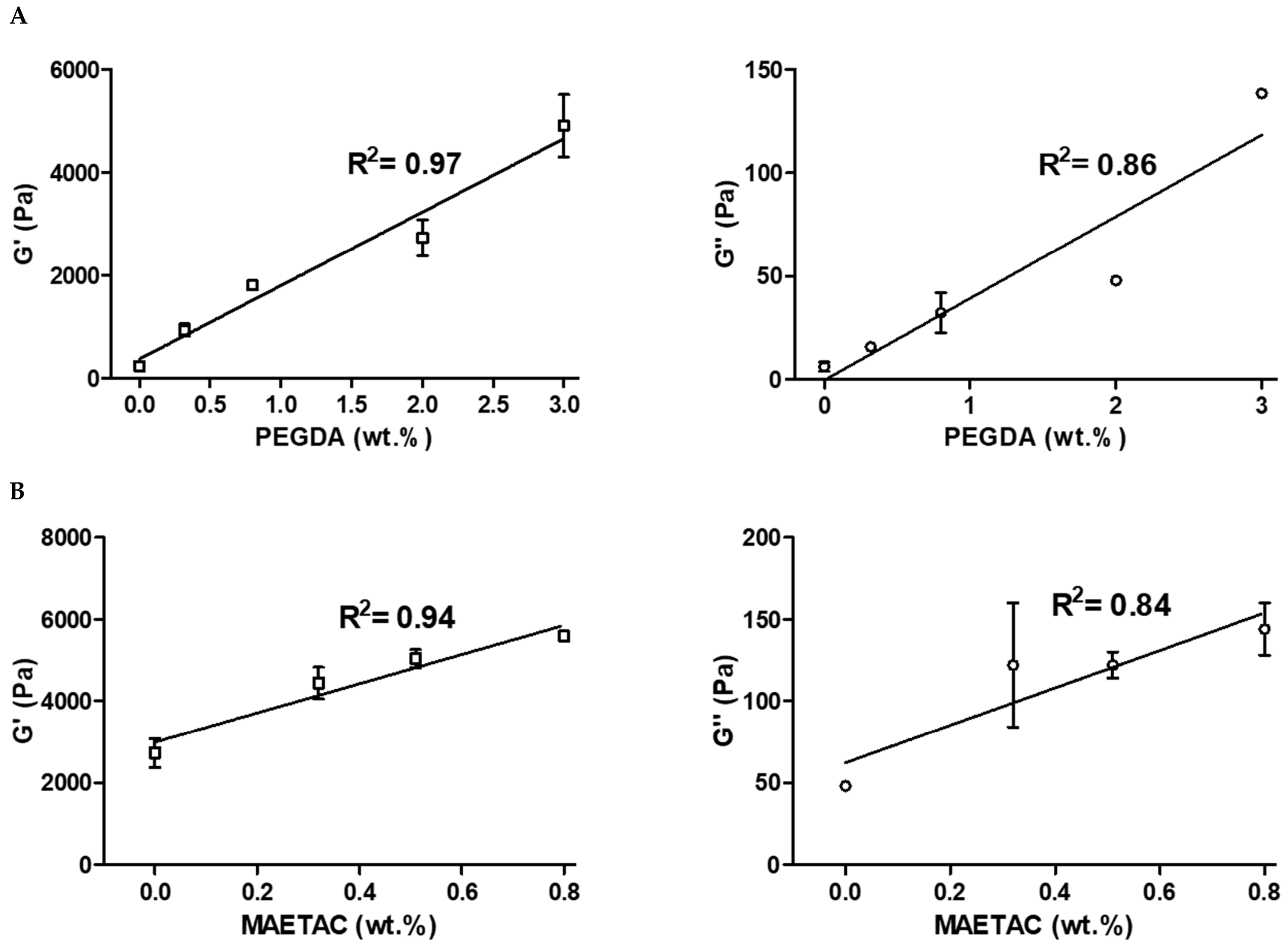

Figure S3. Relationships between G'/G'' moduli of OPF cryogels (OPF 4 wt.%) and amount of PEGDA (A) and OPF/PEGDA cryogels (OPF 4 wt.%, PEGDA 2 wt.%) and amount of MAETAC (B).

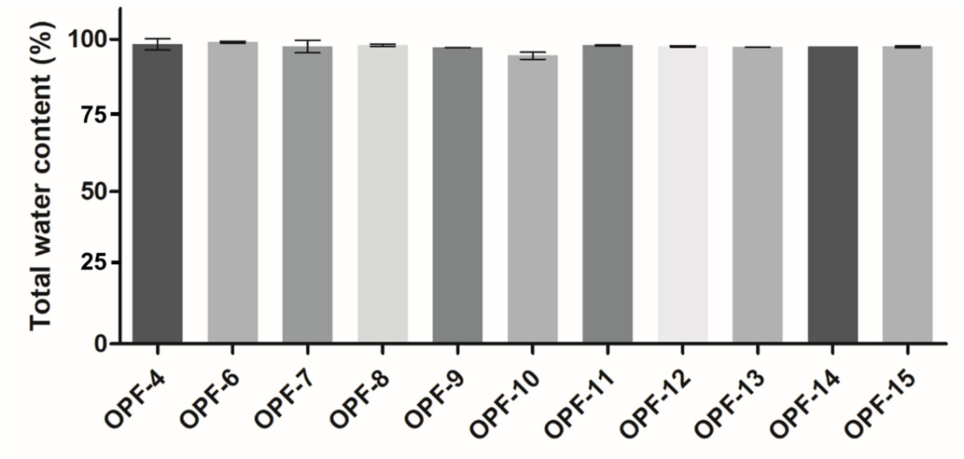

Figure S4. Overall swelling index (total water content %) for OPF-based cryogels.

Group D (OPF 4 %, <u>*PEGDA y%*</u>, MAETAC 0.8%)

OPF-16 (PEGDA 0%)

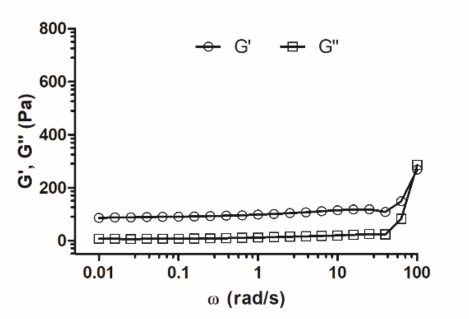

OPF-17 (PEGDA 0.32%)

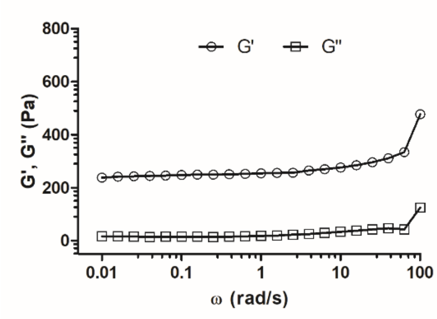

OPF-18 (PEGDA 0.8%)

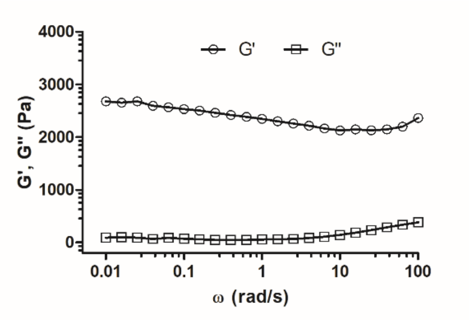

OPF-19 (PEGDA 3%)

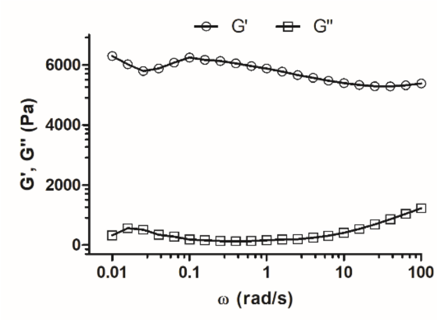

Figure S5. Frequency sweep analysis of OPF-based cryogels (group D). The measurement of frequency dependence of storage (G') and loss (G'') modulus was performed within LVR at δ = 0.5% strain deformation. Cryogel compositions (wt. %) are shown in parentheses.
